# Supplementary material for: Spanish Cross-Cultural Adaptation and Validation of the Oslo Sports Trauma Research Centre (OSTRC) Overuse Injury Questionnaire in Handball Players
Source: Healthcare (Basel). 2023 Mar 21;11(6):912. doi: 10.3390/healthcare11060912 (PMC10048076; doi:10.3390/healthcare11060912)
Supplement: Supplementary file 1 [file healthcare-11-00912-s001.zip › healthcare-2271596-supplementary.pdf]

<u>Supplementary file S1</u> presents the translated and adapted version of the OSTRC (OSTRC-Sp).

Centro de Investigación de traumas deportivos de Oslo (OSTRC)
Cuestionario sobre lesiones por sobreuso.

Parte 1: problemas de rodilla.

Por favor, conteste todas las preguntas, independientemente de si tiene o no problemas con sus rodillas. Seleccione la alternativa que sea más adecuada para usted y, en el caso de que no esté seguro, trate de dar una respuesta lo más adecuada posible de todos modos. El término "problemas de rodilla" se refiere a dolor, rigidez, hinchazón, inestabilidad, bloqueo u otras molestias relacionadas con una o ambas rodillas.

Pregunta 1. ¿Ha tenido alguna dificultad para participar en entrenamientos normales y competiciones debido a problemas de rodilla durante la semana pasada?

□ Participación total sin problemas de rodilla.

□ Participación total, pero con problemas de rodilla.

□ Participación reducida debido a problemas de rodilla.

□ No puedo participar por problemas de rodilla.

Pregunta 2. ¿En qué medida ha reducido su volumen de entrenamiento debido a problemas de rodilla durante la última semana?

□ Sin reducción.

□ En menor medida.

□ Hasta cierto punto.

□ En gran medida.

□ No puedo participar en absoluto.

Pregunta 3. ¿En qué medida los problemas de rodilla han afectado su rendimiento durante la última semana?

□ Sin efecto.

□ En menor medida.

□ Hasta cierto punto.

□ En gran medida.

□ No puedo participar en absoluto.

Pregunta 4. ¿En qué medida ha experimentado dolor de rodilla relacionado con su deporte durante la última semana?

□ Sin dolor

□ Dolor leve

□ Dolor moderado

□ Dolor severo

Parte 2: Problemas lumbares

Responda todas las preguntas independientemente de si tiene o no problemas lumbares. Seleccione la alternativa que sea más adecuada para usted, y en el caso de que no esté seguro, intente dar una respuesta lo mejor posible de todos modos. El término "problemas lumbares" se refiere al dolor, rigidez u otros problemas en la zona lumbar.

Pregunta 1. ¿Ha tenido alguna dificultad para participar en entrenamientos normales y competiciones debido a problemas lumbares durante la semana pasada?

□ Participación total sin problemas lumbares

□ Participación total, pero con problemas lumbares

□ Participación reducida debido a problemas lumbares

□ No puedo participar debido a problemas lumbares

Pregunta 2. ¿En qué medida ha reducido su volumen de entrenamiento debido a problemas lumbares durante la última semana?

□ Sin reducción

□ En menor medida

□ Hasta cierto punto

□ En gran medida

□ No puedo participar en absoluto

Pregunta 3. ¿En qué medida los problemas lumbares han afectado su rendimiento durante la semana pasada?

□ Sin efecto

□ En menor medida

□ Hasta cierto punto

□ En gran medida

□ No puedo participar en absoluto

Pregunta 4. ¿En qué medida ha experimentado dolor lumbar relacionado con su deporte durante la última semana?

□ Sin dolor

□ Dolor leve

□ Dolor moderado

□ Dolor severo

Parte 3: Problemas de hombro

Responda todas las preguntas independientemente de si tiene o no problemas en los hombros. Seleccione la alternativa que sea más adecuada para usted, y en el caso de que no esté seguro, intente dar una respuesta lo mejor posible de todos modos. El término "problemas de hombro" se refiere al dolor, rigidez, flojedad u otras molestias en uno o ambos hombros.

Pregunta 1. ¿Ha tenido alguna dificultad para participar en entrenamientos normales y competiciones debido a problemas en el hombro durante la semana pasada?

□ Participación total sin problemas en el hombro

□ Participación total, pero con problemas en el hombro

□ Participación reducida debido a problemas en el hombro

□ No puedo participar por problemas en el hombro

Pregunta 2. ¿En qué medida ha reducido su volumen de entrenamiento debido a problemas en el hombro durante la última semana?

□ Sin reducción

□ En menor medida

□ Hasta cierto punto

□ En gran medida

□ No puedo participar en absoluto

Pregunta 3. ¿Hasta qué punto los problemas de hombro han afectado su rendimiento durante la última semana?

□ Sin efecto

□ En menor medida

□ Hasta cierto punto

□ En gran medida

□ No puedo participar en absoluto

Pregunta 4. ¿En qué medida ha experimentado dolor en el hombro relacionado con su deporte durante la última semana?

□ Sin dolor

□ Dolor leve

□ Dolor moderado

□ Dolor severo
